# Physical Exercise and Redox Balance in Type 2 Diabetics: Effects of Moderate Training on Biomarkers of Oxidative Stress and DNA Damage Evaluated through Comet Assay

**DOI:** 10.1155/2015/981242

**Published:** 2015-02-19

**Authors:** Monica Pittaluga, Antonio Sgadari, Ivan Dimauro, Barbara Tavazzi, Paolo Parisi, Daniela Caporossi

**Affiliations:** ^1^Department of Movement, Human and Health Sciences, University of Rome “Foro Italico”, 00135 Rome, Italy; ^2^Department of Geriatrics, Gerontology and Physiatrics, University Hospital Agostino Gemelli, Catholic University of the Sacred Heart, 00168 Rome, Italy; ^3^Institute of Biochemistry and Clinical Biochemistry, University Hospital Agostino Gemelli, Catholic University of the Sacred Heart, 00168 Rome, Italy

## Abstract

*Objective.* Hyperglycemia leads to increased production of reactive oxygen species (ROS) in type 2 diabetes, which reduces cellular antioxidant defenses and induces DNA lesions. The aim of this study was to investigate the effects on redox homeostasis and DNA oxidative damage of exercise training in patients with type 2 diabetes compared with nondiabetic individuals. *Methods and Results.* 12 sedentary type 2 diabetic males (62.1 ± 4.3 yrs) and 12 sedentary healthy males (61.7 ± 3.9 yrs) were exposed to 4-month moderate training, 3 times per week, to evaluate the effect on plasma biomarkers of oxidative stress malondialdehyde and antioxidant status (GSSG, GSH/GSSG, and ascorbic acid) as well as basal and H_2_O_2_-induced DNA damage trough alkaline comet assay in peripheral blood lymphocytes. After training, glutathione and ascorbic acid levels increased in both groups, but only in diabetics the malondialdehyde as well as the DNA damage decreased. *Conclusion.* Our study demonstrates for the first time that moderate exercise training is not only effective in improving the redox homeostasis, through an increase of the endogenous antioxidant defences in healthy as well as in diabetic patients, but also, specifically in diabetic patients, effective in lowering the susceptibility to oxidative DNA damage and the lipid peroxidation levels.

## 1. Introduction

Diabetes mellitus (DM) is a metabolic disorder characterized by hyperglycemia resulting from defect either of secretion or action of endogenous insulin [1]. It is one of the most common metabolic diseases since almost 200 million people worldwide are affected, the vast majority having type 2 diabetes mellitus (T2DM) [2–4]. There is considerable evidence suggesting that hyperglycaemia results in the generation of reactive oxygen species (ROS), ultimately leading to oxidative stress in a variety of tissues [5].

An increase of ROS production or the absence of an appropriate compensatory response from the endogenous antioxidants triggers the redox imbalance which plays a key role in the development and progression of DM and its complications [6]. Evidence of depleted antioxidant defences, such as diminished activity of glutathione peroxidase, catalase, and superoxide dismutase, as well as reduced levels of antioxidants in diabetic patients has been widely reported [5–9]. As a consequence, they may accumulate excessively high levels of ROS that lead to acceleration of oxidative damage to cellular proteins, membrane lipids, and DNA [1, 10]. Indeed, in diabetic patients the amount of DNA damage may be correlated with clinical markers [11]: high concentration of 8-hydroxydeoxyguanosine (8-OHdG), a typical product of DNA oxidation and a sensitive marker of oxidative DNA damage [12], was detected in the lymphocytes from diabetic patients [13] and increased levels of DNA breakage were detected in peripheral blood cells from diabetic patients with poor glycemic control [14].

The incidence of T2DM is also associated with environmental factors, among which are the adoption a sedentary lifestyle and an excess of energy intake. Indeed, many evidences show the substantial role of physical activity in the prevention and treatment of diabetes [15, 16]. The relationship between physical activity and decreased diabetes risk have been assessed by a number of prospective studies and clinical trials which indicate that T2DM can be prevented and controlled by regular physical activity [17–19]. Although it is known that the upregulation of antioxidant defences by physical training leads to the overall improvement of cellular response to oxidative-stress, also with respect DNA damage induction and repair [19], and that some studies found a positive effect of training on inflammatory and oxidative patterns linked to diabetic diseases progression and complications [18], human studies analysing the effect of physical training on DNA breakage recurrence in diabetic condition are missing.

### 1.1. Aims

In order to investigate the relationship between physical training (PT) and oxidative stress-related DNA breakage in diabetic patients, we planned a protocol aimed at evaluating the effect of a 4-month PT on two groups consisting of diabetic patients and nondiabetic subjects as controls. We assessed spontaneous and H_2_O_2_-induced DNA damage by single cell gel electrophoresis (SCGE or comet assay) in peripheral blood lymphocytes, controlling plasmatic biomarkers of oxidative stress (malondialdehyde (MDA)) and antioxidant levels (GSSG, GSH/GSSG, and ascorbic acid) before and after the PT.

## 2. Methods

### 2.1. Subjects

The sample consisted of 12 type 2 diabetic patients (DP) (62.1 ± 4.3 years) and 12 healthy males (61.7 ± 3.9 years) as control group (CG). Both the diabetics and the controls were not following any physical training programme at the beginning of the study.

All subjects were recruited at the Department of Geriatrics, Gerontology and Physiatrics, Catholic University of Sacred Heart, Rome. Subjects were completely informed about the study and were asked to sign an informed consent. The study was approved by the Ethical Committee of the Catholic University of Sacred Heart.

Inclusion criteria for the diabetic patients selection were 5 years disease duration at least and therapy with metformin alone or in combination with repaglinide or gliclazide. Exclusion criteria were presence of metabolic unbalance (HbA1c ≥ 7.5), high level neuropathy, high level vasculopathy, cutaneous ulcers, and insulin therapy.

For all subjects, diabetics as well as controls, exclusion criteria were as follows: obesity (BMI ≥ 30), severe cardiovascular diseases (ischemic heart disease or heart failure), chronic obstructive pulmonary disease, severe hypertension (diastolic blood pressure ≥ 100, systolic blood pressure ≥ 200), smoke habits, alcohol abuse, RX treatments, or diagnostics in the 3 months previous to the blood sampling.

After informed consent, blood samples (EDTA or heparin) were collected immediately before and immediately after the 4-months PT. The training sessions were held at the Fitness Center for the Aged, Department of Gerontological, Geriatric and Physiatric Sciences, Catholic University of Sacred Heart. The subjects exercised 3 times per week; each training session consisted of about 30 minutes of callisthenic exercises, followed by 30 minutes of aerobic exercise training at moderate intensity (3–<6 METs; 64–<77% max heart rate) as defined by the ACSM's Guideline for Exercise testing and Prescription [20].

### 2.2. HPLC Separation of Plasma Metabolites

The content of oxidized glutathione (GSSG), reduced glutathione (GSH), MDA, and ascorbic acid was determined in each deproteinized plasma sample by HPLC analysis according to the method detailed as follows. The HPLC method utilized a Kromasil 250 × 4.6 mm, 5 *μ*m particle size column (Eka Chemicals AB, Bohus, Sweden), provided with its own guard column and two buffers with the following composition: 10 mM tetrabutylammonium hydroxide, 10 mM KH_2_PO_4_, 0.25% methanol, pH 7.00 (buffer A); 2.8 mM tetrabutylammonium hydroxide, 100 mM KH_2_PO_4_, and 30% methanol, pH 5.50 (buffer B). A step gradient was obtained as follows: 10 min 100% buffer A; 3 min at up to 90% buffer A; 10 min at up to 70% buffer A; 12 min at up to 55% buffer A; 15 min at up to 45% buffer A; 10 min at up to 25% buffer A; 5 min at up to 0% buffer A. The flow rate of chromatographic runs was 1.2 mL/min and the column temperature was constantly kept at 23°C. The HPLC apparatus consisted of Constametric 2500 pump connected with a SpectraSystem UV6000LP diode array detector (ThermoQuest Italia, Rodano, Milan, Italy) set up between 200 and 300 nm wavelength. Acquisition and analysis of data were performed by a PC provided with the software package (ChromQuest) supplied by HPLC manufacturer. Metabolite concentrations were calculated at 267 nm wavelength (the maximum of MDA absorption) by comparing peak areas of sample runs with those of chromatographic runs of freshly prepared ultrapure standards with known concentrations.

### 2.3. Plasma and Lymphocytes Isolation and Treatments

Plasma was obtained by centrifugation of heparinized blood sample at 800 rpm for 20 min at 4°C. Peripheral blood lymphocytes (PBLs) were isolated by density gradient centrifugation on Ficoll-Paque (Amersham Biosciences). After centrifugation at 1800 rpm for 30 min at room temperature, PBLs were collected from the interface, rinsed twice in phosphate buffered saline (PBS 1X) (EuroClone), and resuspended in RPMI 1640 medium with 2 mM L-glutamine (EuroClone), penicillin (100 U/mL), and streptomycin (0.1 mg/mL) (Sigma-Aldrich). The cells were counted with the trypan blue exclusion test and then split to get four separated cultures (500.000 cells/mL each): 2x untreated (UT) and 2x treated with hydrogen peroxide (H_2_O_2_), 100 *μ*M for 30 minutes.

### 2.4. Comet Assay

To evaluate DNA damage in untreated as well as 100 *μ*M H_2_O_2_ treated PBLs, alkaline (pH > 13) comet assay was performed following the Tice-Vasquez procedure, adapted from the N.P. Singh protocol [21]. Briefly, at the end of treatment, cells were centrifuged for 10 min at 1100 rpm and the pellet was resuspended in PBS. A freshly prepared suspension of cells in 0.75% low melting point agarose (LMA Sigma Chemicals) dissolved in PBS w/o Ca++ and Mg++ was cast onto microscope slides precoated with 0.5% normal melting point agarose (NMA Sigma Chemicals). The cells were then lysed for 1 h at 4°C in a lysis buffer consisting of 2.5 M NaCl, 100 mM EDTA, 1% Triton X-100, 10% DMSO, and 10 mM Tris, pH 10. After the lysis, DNA was allowed to unwind for 40 min in electrophoretic solution consisting of 300 mM NaOH and 1 mM EDTA, pH > 13. Electrophoresis was conducted at 4°C for 30 min at electric field strength of 0.73 V/cm (30 mA). The slides were then neutralized with 0.4 M Tris, pH 7.5, fixed with methanol, and stored at RT. The slides were stained with 100 *μ*L 1X ethidium bromide and immediately examined at 20x magnification in an Eclipse fluorescence microscope (Nikon, Tokyo, Japan) attached to a COHU 4910 video camera (Cohu, Inc., San Diego, CA, USA) and connected to a personal computer-based image analysis system, Komet 5.5 Image Analysis System (Kinetic Imaging Ltd, Liverpool, UK). Fifty images were randomly selected from each sample and the comet tail DNA was measured. Two parallel tests with aliquots of the same sample of cells were performed for a total of 100 cells. Each experiment was repeated two times.

Percentage of DNA in the tail (% tail DNA) was analyzed. It is positively correlated with the level of DNA breakage or/and alkali labile sites in the cell. The mean value of the % tail DNA in a particular sample was taken as an index of DNA damage in this sample. Statistical analysis of the comet data was performed by calculation of median of each experimental point and then analyzing the differences between the means of the median values clustered by treatment.

### 2.5. Data Analysis

Collected data were organized in a database and analyzed with the statistic software SPSS (SPSS 15 for Windows). The results are expressed as mean ± standard error of the mean (SEM). Statistical significance was set to *P* < 0.05. All the data were tested for their normal distribution with the one–sample Kolmogorov-Smirnov test.

Paired *t*-test was used to analyze the differences between subjects belonging to the same group before and after PT. The *t*-test for independent sample was applied to analyze the differences between diabetic patients and control group.

## 3. Results

### 3.1. Clinical Data

As expected, after PT, the glycemic control resulting significantly improved in diabetic patients, as revealed by the decrease of both basal glycemia (*P* = 0.023) and glycated haemoglobin (*P* = 0.01). In addition, PT decreased the body mass index (BMI) in both diabetic patients (*P* = 0.001) and control group (*P* = 0.01) (Table 1).

### 3.2. Redox Homeostasis

**Figure 1:**
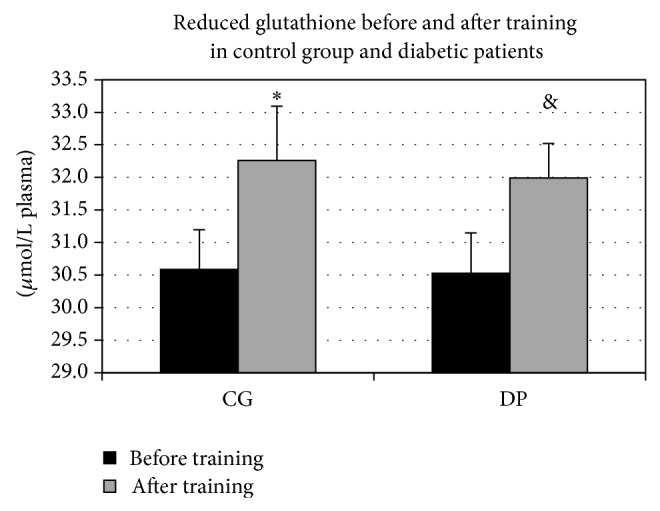
Reduced glutathione before and after training in control group and diabetic patients. After training, reduced glutathione increased both in control group (CG) (^*^ *P* = 0.015) and in diabetic patients (DP) (^&^ *P* < 0.0001).

**Figure 2:**
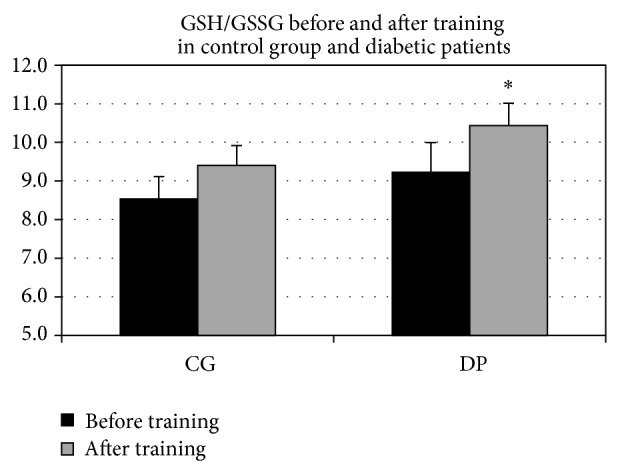
GSH/GSSG before and after training in control group and diabetic patients. After training only diabetic patients (DP) showed an increase in reduced/oxidized glutathione ratio (^*^ *P* = 0.04).

The response of biochemical parameters to PT, both in diabetic patients (DP) and in control group (CG), showed significant differences. Reduced glutathione increased in DP (GSH baseline mean value: 30.53 ± 2.05; GSH PT mean value: 31.99 ± 1.67; *P* < 0.0001) as well as in CG (GSH baseline mean value: 30.58 ± 2.21; GSH PT mean value: 32.26 ± 2.20; *P* = 0.015) (Figure 1), but only in DP a significant increase of glutathione ratio (GSH/GSSG) was observed (*P* = 0.042) (GSH/GSSG baseline values: DP 9.22 ± 2.56; CG 8.53 ± 2.07; GSH/GSSG PT values: DP 10.44 ± 1.82; CG 9.41 ± 1.35) (Figure 2). No significant differences were found between the DP and CG basal values for both reduced glutathione and glutathione ratio.

**Figure 3:**
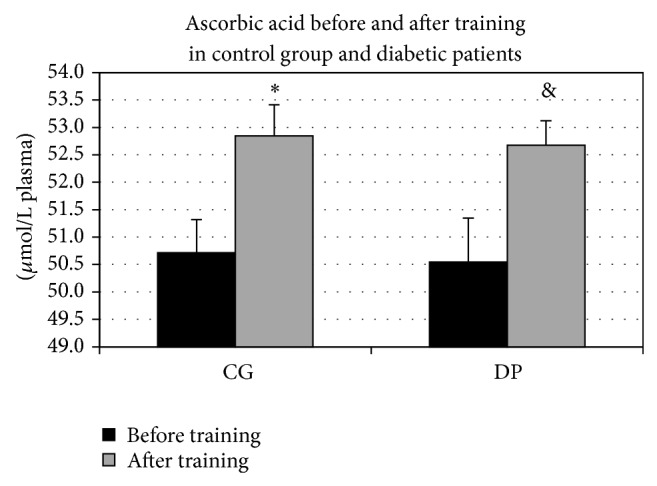
Ascorbic acid before and after training in control group (CG) and diabetic patients (DP). Ascorbic acid (AA) significantly increased after training both in CG (^*^ *P* = 0.016) and in DP (^&^ *P* < 0.0001).

The plasma concentration of ascorbic acid (AA), significantly increased after PT both in controls (*P* = 0.016) and in diabetic patients (*P* < 0.0001) (Figure 3), whereas no differences were seen in AA basal values between diabetic patients and control group.

### 3.3. Oxidative Damage

**Figure 4:**
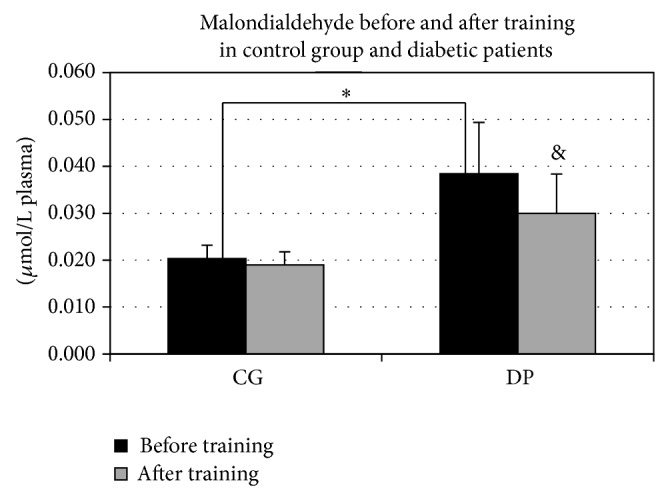
Malondialdehyde before and after training in control group and diabetic patients. Malondialdehyde was reduced after training in diabetic patients (DP) (^&^ *P* = 0.02). A significant difference was showed between the two groups before training, the level of DP being higher compared to controls (^*^ *P* = 0.014).

A significant difference was shown between CG and DP in the baseline levels of malondialdehyde (MDA), the level of DP being higher compared to controls (MDA baseline mean values: CG versus DP; *P* = 0.014) (Figure 4). After training, the MDA values showed a very similar behavior to that of GSH/GSSG ratio, being significantly reduced by PT only in DP and not having any effect in CG (CG: MDA baseline versus MDA PT *P* = 0.75; DP: MDA baseline versus MDA PT *P* = 0.02).

**Figure 5:**
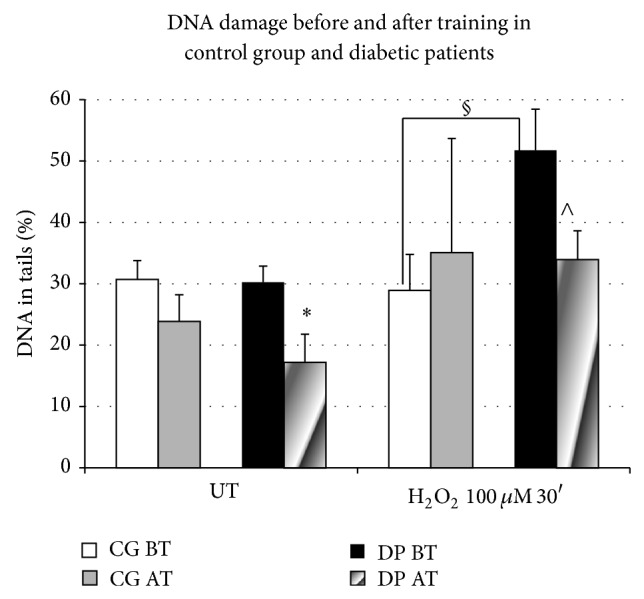
% of DNA in tails in untreated and H_2_O_2_-treated PBLs before training (BT) and after training (AT) in control group (CG) and diabetic patients (DP). DP showed a significant diminution in % DNA in tails in untreated samples (UT) (^*^ *P* = 0.02). In the H_2_O_2_ treated samples (H_2_O_2_, 100 uM × 30′), the percentages of DNA in tails were different between DP and CG before training (^§^ *P* = 0.049), and a significant diminution after training in the DP nuclei (^∧^ *P* = 0.04) was observed.

With regard to the DNA damage detected by comet assay in PBLs, no differences were detected in the spontaneous rates between CG and DP before training (BT) in untreated (UT) PBLs (*P* = 0.91, % DNA in tails in UT ranging between 16.42 and 49.65). After training (AT), lymphocytes from both groups showed a decrease in the % DNA in tails of untreated PBLs, being significant only in diabetic patients (*P* = 0.02) (Figure 5). In H_2_O_2_ treated samples, we observed a significant difference between diabetic subjects and controls in the total % DNA in tails (CG1 BT versus DP1 AT: *P* = 0.049) (Figure 5), as well as a decrease of total % DNA in tails only in the diabetes group after training (DP1 BT versus DP1 AT: *P* = 0.04). To evaluate the effect of training on the H_2_O_2_-induced DNA damage, the Δ values for the net increase of % tail DNA after the H_2_O_2_ treatment was analyzed (% DNA tail in H_2_O_2_ treated; % DNA tail in untreated). This analysis confirmed the higher susceptibility of diabetes patients to the induction of DNA damage by oxidative insult (DP versus CG, *P* < 0.05), while the H_2_O_2_-induced DNA damage in DP after training did not show statistical differences with respect the BT or CG Δ values (*P* > 0.05) (Table 2).

## 4. Discussion

### 4.1. Clinical Results

Both the metabolic control and the energy balance, indicated by a diminution of body mass, were improved by the regular PT, confirming the well-known role of physical activity as powerful aid to antidiabetic drugs in controlling the metabolic balance in diabetic patients [22].

### 4.2. Plasmatic Redox Balance

Our biochemical data confirmed the hypothesis that training can cause adaptive responses in terms of endogenous antioxidant, as well as an improvement in ascorbic acid recycling mechanisms. The results showed that training positively affected the antioxidant serum content, as evaluated through the increment of GSH, both in diabetic patients and controls. Our findings are in agreement with previous research demonstrating that glutathione is a powerful redox sensitive marker, able to detect changes induced by regular exercise [23]. Changes in the glutathione system are usually assessed because GSSG efflux from cells into the plasma is considered indicative of oxidative stress and recent evidences show its relation to the muscle ROS production [24]. GSH is oxidized to GSSG in cells in response to an increase in free radicals. When the rate of oxidation is low, much of the GSSG produced may be enzymatically reduced by GSSG reductase activity to GSH. However, with a more severe oxidative stress, the rate of GSSG reduction cannot match that of its formation, thus resulting in the accumulation of intracellular GSSG.

Recent findings confirm the specific action of PT on glutathione metabolism in type 2 diabetes [25]. The finding that only in diabetic patients the GSH/GSSG ratio increased significantly after training could be ascribed to the facts that type 2 diabetes condition implies a higher susceptibility to oxidization, as is showed by malondialdehyde plasma concentration (see ahead). Thus, the reduction of the oxidative products after training could be more incisive and detectable in this group compared with unaffected subjects.

In our sample, exercise training also reduced plasma lipid oxidation and peroxidation, which is expected to be higher in diabetic patients. An increased level of lipid peroxidation in leucocytes from patients with T2DM has been reported [10]. Moreover, an enhanced production of MDA, a marker of lipid peroxidation, has been demonstrated in the erythrocyte membranes from diabetic patients, and decrease of reduced glutathione as well as ascorbic acid levels was found in leucocytes from patients with T2DM [12]. The results from the MDA analysis indicated that this higher susceptibility to oxidative damage may be improved by a moderate PT. These findings support that line of research, which demonstrated that a moderate PT may be effective in blunting lipid peroxidation, as well as in other oxidative stress markers and inflammation [26–28]. These results are associated with and probably due to decreased hyperglycaemia that is possibly also the main cause of the beneficial effects, considering that it is obtained also by controlling the dietary intake [29]. It is well known how a long term hyperglycaemic condition eventually leads to unbalance between antioxidant and prooxidant components. In particular, ascorbic acid is relevant for the insulin secretion. In the light of this, the outcome of our research appears very important, considering the significant increment that ascorbic acid levels have shown in both groups after training. As the lifestyle conditions were not changed during the study period, we can argue that this increment is not due to a change in diet habits but probably in an upgrading of the ascorbic acid recycling efficiency.

### 4.3. DNA Damage

With regard to the DNA damage detected by comet assay, we did not detect any significant difference between control and type 2 diabetes groups in the baseline levels of % DNA in tails. Similar result has been shown by Ibarra-Costilla et al. who found similar values for the length of tail comet and tail extent moment in blood cells from three age-related groups of patients with type 2 diabetes and the control group [30]. Conversely, a higher level of oxidative DNA damage measured by the comet assay in leukocytes obtained from diabetic patients compared to healthy subjects has been described in the literature [11, 31, 32], where it is also verified that the increase in DNA damage correlates with very poor glycemic control and lower antioxidant capacity [31]. Considering that the T2DM patients in this study showed a stabilized glycemic control with a baseline antioxidant capacity similar to those detected in the control group, our data further verify that, beside the diabetics condition, the increase in PBLs DNA damage mainly depends upon oxidative stress driven by high glycaemia. Concerning the hydrogen peroxide treated samples, our data confirmed the increased susceptibility to oxidative DNA damage [33, 34], the percentage of DNA in tails in diabetic patients being significantly higher in comparison with controls, as measured by both total and net increases of % DNA in tails. After the training, only the spontaneous % DNA in the tails of diabetic patients resulted positively modified, while no significant effect could be detected with respect the H_2_O_2_-induced damage. The single cell gel electrophoresis (SCGE) or comet assay in peripheral blood lymphocytes represents the method of choice for measuring DNA damage from exposure to genotoxic agents including radiation, chemicals, and oxidative stress [35, 36]. The DNA damage detected by alkaline comet assay represents a steady state between induction of lesions and their repair, detectable in both proliferating and nonproliferating cells, being considered a reliable biomarker of exposure to ROS-induced damage [37].

Although our study cannot exclude the possibility of differences in DNA repair efficiency between control and diabetes groups [38], the results from the comet assay combined with our results on redox status suggest that the diminution in spontaneous DNA damage observed after training is probably ascribable to a decrease of the prooxidant cellular conditions. The beneficial action of moderate PT on the cellular redox balance is confirmed by literature and more recently it has been showed how a moderate and long lasting exercise may act as a stimulus toward mitochondrial adaptation, particularly at muscular level, thus reversing the mitochondrial impairment seen in diabetic condition and the consequent ROS overproduction [39].

## 5. Conclusion

In line with the recent literature on the beneficial effect of physical exercise in the prevention and therapy of T2DM, our study demonstrated that four months of moderate PT are effective in improving the redox homeostasis in healthy as well as in diabetic patients, probably lowering the oxidant species production and/or increasing the endogenous antioxidant defences. Moreover, this research showed for the first time that physical exercise is able to lower the oxidative DNA damage in PBLs from type 2 diabetics as measured by SCGE or comet assay.

## Figures and Tables

**Table 1:** Clinical parameters before and after physical training.

	Diabetic patients			Control group		
	Before training	After training	*P* value	Before training	After training	*P* value
			(paired *t*-test)			(paired *t*-test)
BMI (kg/m^2^)	28.3 ± 0.6	27.9 ± 0.7	0.001	30.2 ± 0.8	29.7 ± 0.8	0.01
BG (mg/dL)	116.3 ± 3.6	106.4 ± 1.9	0.02	87.8 ± 2.2	90.3 ± 3.0	ns
HbA1c (%)	6.7 ± 0.2	6.3 ± 0.1	0.01	5.5 ± 0.1	5.4 ± 0.1	ns

BMI: body mass index; BG: basal glycemia; HbA1c: glycated haemoglobin.

**Table 2:** H_2_O_2_-induced % tail DNA in PBL (mean Δ values ± SEM) from subjects in control group (CG) and type 2 diabetic patients (DP).

	Before training	After training	*P* value
			(paired *t*-test)
CG	−2.50 ± 4.97	11.8 ± 9.29	0.21
DP	35.94 ± 11.16	15.84 ± 5.03	0.17
*P* value	0.033	0.32	
(paired *t*-test)			
